# Proteomic and Bioinformatic Analysis of *Streptococcus suis* Human Isolates: Combined Prediction of Potential Vaccine Candidates

**DOI:** 10.3390/vaccines8020188

**Published:** 2020-04-18

**Authors:** Esther Prados de la Torre, Antonio Rodríguez-Franco, Manuel J. Rodríguez-Ortega

**Affiliations:** Departamento de Bioquímica y Biología Molecular, Universidad de Córdoba, and Campus de Excelencia Internacional CeiA3, 14071 Córdoba, Spain

**Keywords:** *Streptococcus suis*, zoonosis, human infection, proteomics, surface proteins, “shaving”, reverse vaccinology, protein vaccine candidates

## Abstract

*Streptococcus suis* is a Gram-positive bacterium responsible for major infections in pigs and economic losses in the livestock industry, but also an emerging zoonotic pathogen causing serious diseases in humans. No vaccine is available so far against this microorganism. Conserved surface proteins are among the most promising candidates for new and effective vaccines. Until now, research on this pathogen has focused on swine isolates, but there is a lack of studies to identify and characterize surface proteins from human clinical isolates. In this work, we performed a comparative proteomic analysis of six clinical isolates from human patients, all belonging to the major serotype 2, by “shaving” the live bacterial cells with trypsin, followed by LC-MS/MS analysis. We identified 131 predicted surface proteins and carried out a label-free semi-quantitative analysis of protein abundances within the six strains. Then, we combined our proteomics results with bioinformatic tools to help improving the selection of novel antigens that can enter the pipeline of vaccine candidate testing. Our work is then a complement to the reverse vaccinology concept.

## 1. Introduction

*Streptococcus suis* is a Gram-positive bacterium which inhabits as a commensal in the upper respiratory tract of pigs, colonizing up to 100% of the animals [1]. However, it can cause severe infections such as bronchopneumonia in the lower respiratory system of swine, as well as invasive diseases including meningitis, endocarditis, sepsis, and even sudden death [2,3]. Therefore, in addition to its impact in animal welfare, the economic importance of this pathobiont species is very high, as it is responsible for monetary losses in the livestock industry worldwide, increasing also the cost of production because of supplying prophylactic antibiotics [4].

In addition, *S. suis* is considered an emerging zoonotic pathogen, causing infections in humans that are in contact with infected pigs, mainly in the slaughter industry, as well as in people consuming raw or poorly cooked pork meat, or other pork byproducts [5,6,7]. Two outbreaks in 1998 and 2005 leading to high mortality rates caused numerous human casualties in China [1,3,8], and has become endemic in other South-East Asian countries. In Vietnam and Thailand, *S. suis* infections are amongst the most common causes of meningitis in adults [4,8,9]. Additionally to Asia, many other cases of infections in humans have also been reported in Europe, America, and Oceania [8].

*S. suis* strains are classified in 35 different serotypes according to their serological reaction of the capsular polysaccharide [10]. Of these, serotype 2 (SS2) is by far the most prevalent worldwide, being highly virulent both in pigs and in humans. Whereas there are differences in the geographical prevalence of serotypes in animals, for example, SS9 is predominant in Europe, while SS2 prevails in many other regions in the world, the vast majority of *S. suis* infections in humans are associated with SS2 [8,10]. There is no effective commercial vaccine to prevent infections caused by this pathogen, although several approaches have been attempted, including the use of bacterins or live-attenuated strains [11,12,13]. However, most efforts were made in the last years to develop a protein subunit-based vaccine, which can confer cross-protection against all, or at least, the most prevalent and virulent serotypes. For that purpose, surface proteins are the most interesting candidates for the development of protein vaccines, as they have the highest chance to raise an effective immune response [14,15]. To date, all research has been conducted to develop a vaccine against *S. suis* infecting pigs; there is thus a lack of research concerning vaccines against those isolates affecting humans.

Proteomics offers the possibility to identify many proteins in a single analysis, using adequate platforms [16,17]. In the field of infectious diseases, proteomics can be used to identify and characterize in a fast and reliable way sets of surface proteins (known as the “surfome” or “surfaceome”) by “shaving” live microorganism cells with proteases, followed by LC-MS/MS analysis of the recovered peptides [18,19,20,21,22,23,24]. Thus, when comparing a large set of strains of a given pathogen, the obtained “pansurfome” may provide reasonably good protein vaccine candidates with potential cross-protection, based on their abundance and distribution in the studied strains [20,25,26]. We previously defined the “pansurfome” of a large collection of *S. suis* swine isolates for candidate selection [25], as well as the “immunosecretome” to propose alternative candidates from secreted proteins [27]. In this work, and for the first time, we carried out a proteomic comparison of six SS2 human isolates and, supported by bioinformatic inspection, we propose a list of proteins and/or fragments with predicted antigenicity that can enter future pipelines to develop vaccines to prevent infections by this pathogen in humans. Thus, this work represents a step beyond classical reverse vaccinology [28,29], using proteomics to experimentally support only genome-based antigen prediction that can help drive efforts towards more accurate potential vaccine candidates (PVCs), based on empirical information.

## 2. Materials and Methods

### 2.1. Bacterial Strains and Culture Conditions

*S. suis* strains used in this study, all belonging to the major serotype 2, were isolated from diseased human patients suffering meningitis (Table 1). All the strains were kept at −80 °C in vials containing 15% glycerol until use. For further “shaving” experiments, they were plated on Columbia agar blood base that contained 6% (*v*/*v*) sheep blood, and then grown in Todd–Hewitt broth at 37 °C and 5% CO_2_ until reaching an OD_600_ = 0.25, corresponding to the mid-exponential phase.

### 2.2. Bacterial Surface “Shaving” and Peptide Extraction

Peptides from surface proteins were obtained by the bacterial “shaving” approach as already described [25,30], with slight modifications. Briefly, 25 mL of cultures were centrifuged at 3500× *g* for 10 min at 4 °C, and the pelleted bacteria were washed twice with phosphate-buffered saline (PBS). Cells were resuspended in 0.5 mL PBS/30% sucrose (pH 7.4). Protease “shaving” of resuspended bacteria was performed with 1 µg trypsin (Promega, Madison, WI, USA) for 30 min at 37 °C with top-down agitation within an incubator. The resulting digestion mixtures were centrifuged again at 3500× *g* for 10 min at 4 °C, and the supernatants, which contained the peptides (i.e., the ‘‘surfome’’ fractions) were filtered using 0.22 µm pore-sized filters (Merck-Millipore, Burlington, MA, USA). Surfomes were re-digested with 0.5 µg trypsin overnight at 37 °C with top-down agitation. Peptides were purified prior to analysis, using Oasis HLB extraction cartridges (Waters, Milford, MA, USA). Peptides were eluted with increasing concentrations of acetonitrile (ACN)/0.1% formic acid, according to the manufacturer’s instructions. Peptide fractions were concentrated with a vacuum concentrator (Eppendorf, Hamburg, Germany), resuspended in 100 µL of 2% ACN/0.1% formic acid, and kept at −20 °C until further analysis.

### 2.3. LC-MS/MS Analysis

Peptide separation was performed by nano-LC using a Dionex Ultimate 3000 nano UPLC (Thermo Scientific, San Jose, CA, USA), equipped with a reverse phase C18 75 μm × 50 Acclaim Pepmap column (Thermo Scientific) at 300 nL/min and 40 °C for a total run time of 85 min. The mix of peptides was previously concentrated and cleaned up on a 300 μm × 5 mm Acclaim Pepmap cartridge (Thermo Scientific) in 2% ACN/0.05% formic acid for 5 min, with a flow of 5 µL/min. Solution A (0.1% formic acid) and solution B (80% ACN, 0.1% formic acid) were used as mobile phase for the chromatographic separation according to the following elution conditions: 4–35% solution B for 60 min; 35–55% solution B for 3 min; 55–90% solution B for 3 min followed by 8 min washing with 90% solution B, and re-equilibration for 12 min with 4% solution B.

Peptide positive ions eluted from the column were ionized by a nano-electrospray ionization source and analyzed in positive mode on a trihybrid Thermo Orbitrap Fusion (Thermo Scientific) mass spectrometer operating in Top30 Data Dependent Acquisition mode with a maximum cycle time of 3 s. Single MS scans of peptide precursors were acquired in a 400–1500 *m*/*z* range at 120,000 resolution (at 200 *m*/*z*) with a 4 × 10^5^ ion count target threshold. For MS/MS, precursor ions were previously isolated in the quadrupole at 1.2 Da, and then CID-fragmented in the ion trap with 35% normalized collision energy. Monoisotopic precursor selection was turned on. Ion trap parameters were: (i) the automatic gain control was 2 × 10^3^; (ii) the maximum injection time was 300 ms; and (iii) only those precursors with charge state 2–5 were sampled for MS/MS. In order to avoid redundant fragmentations a dynamic exclusion time was set to 15 s with a 10-ppm tolerance around the selected precursor and its isotopes.

### 2.4. Protein Identification and Database Searches

The mass spectrometry raw data were processed using Proteome Discoverer (version 2.1.0.81, Thermo Scientific). Charge state deconvolution and deisotoping were not performed. MS/MS spectra were searched with SEQUEST engine (version v.27, Thermo Scientific) against a local database containing all the proteins derived from the genome sequence of *Streptococcus suis* BM407 (downloaded from [31], and applying the following search parameters: Trypsin was used for theoretical digestion of protein sequences, allowing up to one missed cleavage. Methionine oxidation was set as variable modification. A value of 10 ppm was set for mass tolerance of precursor ions, and 0.1 Da tolerance for product ions. Peptide identifications were accepted if they exceeded the filter parameter Xcorr score versus charge state with SequestNode Probability Score (+1 = 1.5, +2 = 2.0, +3 = 2.25, +4 = 2.5). Validation of peptide spectral matches (PSM) was done at a 1% false discovery rate (FDR) using a percolator based on q-values. For protein quantification, precursor ion areas were calculated using the precursor ion area detector and normalized by the total protein amount mode in Proteome Discoverer 2.1.

### 2.5. Computational Prediction of Protein Subcellular Localization

Primary predictions of *S. suis* BM407 protein subcellular localization were assigned by using the web-based algorithm LocateP v2 [32]. They were contrasted by several feature-based algorithms: TMHMM 2.0 [33] for searching transmembrane helices; SignalP 5.0 [34] for type-I signal peptides; LipoP 1.0 [35] for identifying type-II signal peptides, which are characteristic of lipoproteins. Kyoto Encyclopedia of Genes and Genomes (KEGG) Orthology (KO) annotations were retrieved from the KEGG database and were used for inferring relationships using the KEGG Mapper suite [36]. In silico prediction of protein vaccine candidates and antigens was done using the web-based algorithm VaxiJen 2.0 [37].

### 2.6. Data and Statistical Analysis

Peptide extractions were made in triplicate from three independent cultures and “shaving” experiments for each strain. Proteins were considered to be present in a given sample as long as they were identified in at least two out of the three biological replicates for such a sample. Otherwise, proteins found only in one biological replicate were not considered to be found in the sample(s) and were discarded from the overall count of identified proteins. For further quantitative analysis, means and standard deviations were calculated using an Excel spreadsheet (Microsoft Excel 2011 v14.0.0 for Mac, Microsoft, Redmond, WA, USA). Values were z-scored prior to the principal component and clustering analysis. The R package FactoMineR was used to analyze the data mainly through principal component analysis. The factoextra package was used to represent these analyses, and the pheatmap package to cluster the data and represent the corresponding heatmaps. Non-detected proteins in samples were assigned a 0 value to avoid the processing of not available (NA) data.

## 3. Results

### 3.1. Bacterial Surface “Shaving” and Protein Identification

We analyzed six human clinical isolates from different and distant provinces of Spain. All of them suffered from meningitis. Three strains were isolated from cerebrospinal fluid (CSF), and the other three from blood. The six isolates were SS2.

After “shaving” the six SS2 isolates with trypsin and further LC-MS/MS analysis, the MS/MS spectra were searched against the human SS2 reference strain BM407. From the total of 557 surface proteins predicted by the LocateP v2 prediction algorithm, 131 surface proteins were identified in the six isolates (i.e., the “pan-surfome” of these strains), grouped in the following categories (Table 2): 31 signal peptide II lipoproteins, out of 40 from this category predicted from the BM407 genome (i.e., 77.5%); 18 LPXTG cell wall-anchoring proteins, out of 20 predicted in the reference strain, representing 90% of all the proteins in this category; 11 out of 18 secreted proteins (i.e., proteins with signal peptide I), representing 61.1% of all predicted secretory proteins; and finally, 71 proteins with one or more transmembrane domains (TMD), out of the 557 predicted from the genome of this reference strain (i.e., 23.5% of total membrane proteins). Of these, 45 possessed only 1 TMD (110 predicted in the BM407 genome, i.e., 40.9%), and 26 were multi-transmembrane proteins (i.e., proteins with more than 1 TMD). These represented only 7.1% of the predicted multi-transmembrane proteins (369 in total in the genome). In addition, 759 proteins predicted as cytoplasmic were identified, but they were excluded because this work was focused on those proteins predicted to be at the extracellular side of the bacterial cell.

Table 3 shows the complete list of the 131 identified surface proteins, as well as their presence in each individual SS2 isolate. As expected, the very vast majority of lipoproteins and predicted secreted proteins were identified in all the isolates. The same occurred with cell wall proteins, with some exceptions: two putative glucan-binding surface-anchored proteins, encoded by loci *SSUBM407_0471* and *SSUBM407_0949*, were found only in the 1086/11 strain. However, this was not accomplished in the case of membrane proteins: those with 1 TMD were more frequently found in all (or most of the) isolates, but those with more than 1 TMD were more scarcely identified. Actually, only four in this last subcategory were found in the six clinical isolates: SSUBM407_0896, SSUBM407_1298, SSUBM407_1682, and SSUBM407_1747.

Interestingly, among the LPXTG cell wall proteins, we identified the major pilus protein encoded by the locus *SSUBM407_0414*. Pili proteins have been shown previously to be trypsin resistant [21,38,39], but here the protein identified was found in the six clinical isolates analyzed, with 30 peptides covering 54% of the protein sequence in its immature form (Figure S1), or 61% of the mature protein sequence (once *N*-term signal peptide and *C*-term sortase post-processing sequence are removed).

### 3.2. Analysis of Differences in Surface Protein Abundances among the Clinical Isolates

**Figure 1:**
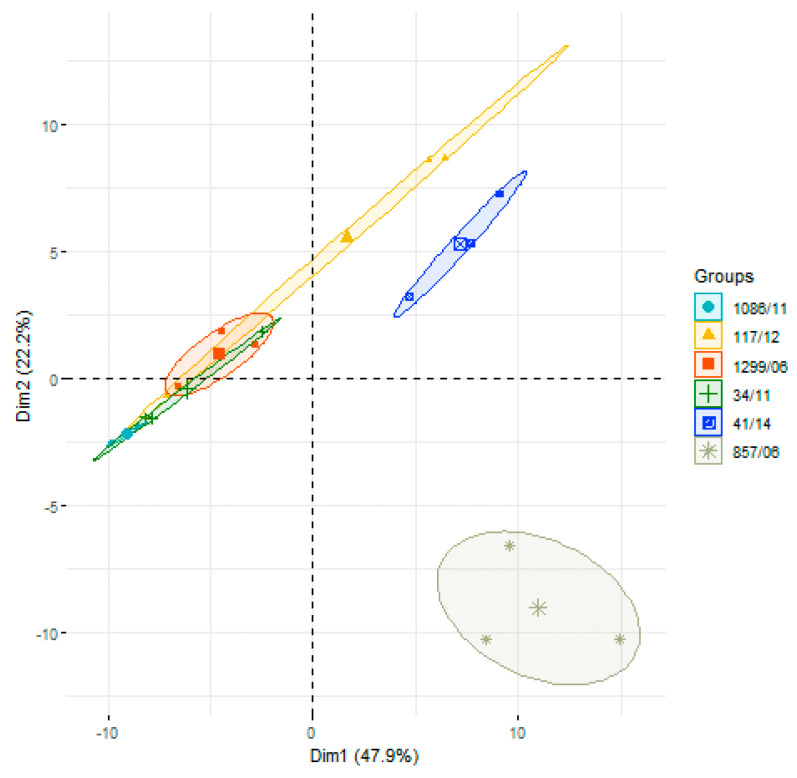
Principal component analysis (PCA) of global surface proteins identified in the six *Streptococcus suis* human clinical isolates.

Next, after protein identification, we performed a label-free semi-quantitative analysis to determine differences in the abundances of surface proteins among the six clinical isolates, based on chromatography peak areas (Table S1). For that, we first carried out a principal component analysis (PCA) to evaluate differences in the overall pattern of surface protein abundances comparing the six isolates (Figure 1). The two first dimensions of the analysis explained 70.1% of the variance, with principal component (PC) 1 responsible for 47.9%, and PC2 for 22.2%. In general, the three biological replicates of each strain were well grouped, except for isolate 117/12 in which replicate #1 showed a great dispersion from the other two replicates, as measured by the Euclidean distances (Figure S2). The PCA showed that strains 857/06 and 41/14 were clearly separated from the other four isolates, and that 1299/06 and 34/11 were quite close each other, with 1086/11 separated from 1299/06 and partially overlapping with 34/11. The isolate 117/12 could also constitute a clearly differentiated group from the rest, but the dispersion due to the distance of replicate #1 from the other two made this strain partly overlap with the group formed by isolates 1299/06, 34/11, and 1086/11.

**Figure 2:**
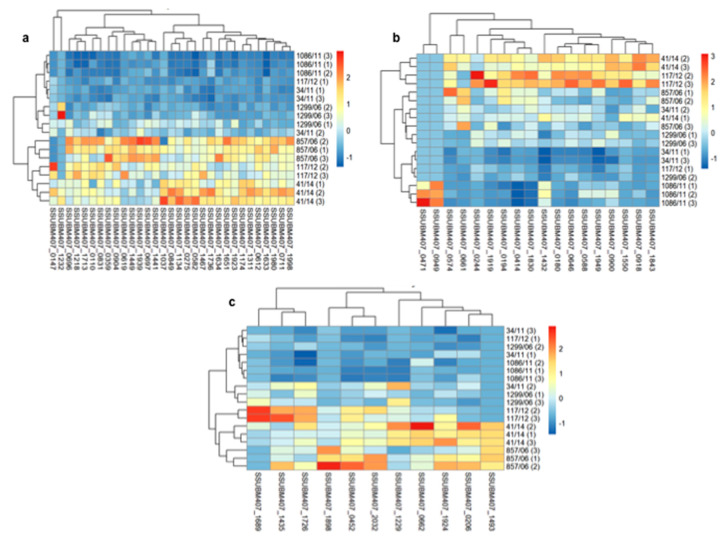
Hierarchically-clustered heatmaps of z-scored surface protein abundances in the six *Streptococcus suis* human clinical isolates. Proteins are clustered in columns in each heatmap, and isolates in rows. The numbers in parentheses in clinical isolates represent each of the three biological replicates. (**a**) Lipoproteins; (**b**) cell wall proteins; (**c**) secreted proteins.

**Figure 3:**
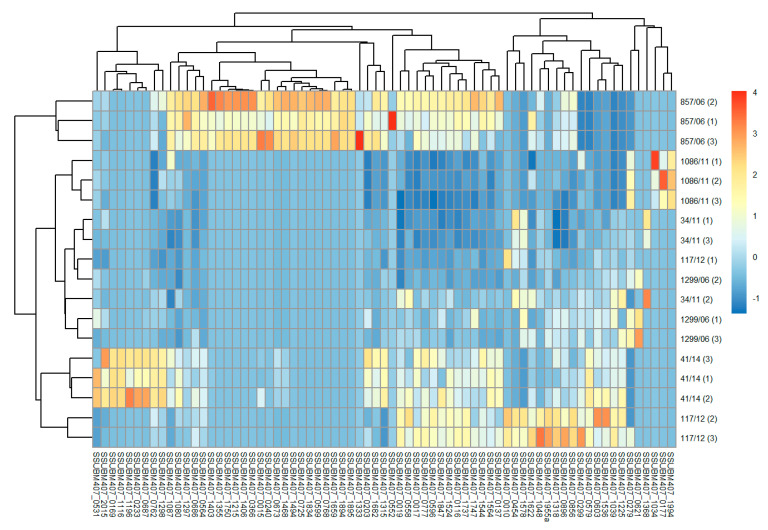
Hierarchically-clustered heatmaps of z-scored abundances of predicted membrane proteins in the six *Streptococcus suis* human clinical isolates. Proteins are clustered in columns in each heatmap, and isolates in rows. The numbers in parentheses in clinical isolates represent each of the three biological replicates.

Then, in hierarchically-clustered heatmaps, we presented the z-score abundances of the 131 identified surface proteins, grouped in four major categories of subcellular localization: lipoproteins, cell wall proteins, secreted, and membrane proteins. In general terms, it can be appreciated that, for most proteins, there was a higher expression on the surface of isolates 857/06, 41/14, and 117/12 compared to the other three strains, although this tendency varied according to particular proteins and subcellular localization category. Thus, for lipoproteins, the highest abundances of most proteins were found in strains 857/06, 41/14, and 117/12 (Figure 2a). The highest abundance levels of LPXTG-cell wall proteins were found in 41/14 and 117/12, followed by 857/06 (Figure 2b). However, in this category there was a greater dispersion of values between replicates. Of note, as stated before, the 1086/11 strain was the only one to express the two putative glucan-binding surface-anchored proteins SSUBM407_0471 and SSUBM407_0949. For most of the 11 identified secreted proteins, higher abundances were found in 857/06 and 41/14, and to a lesser extent, in 117/12, compared to the other three isolates (Figure 2c). Finally, the category of membrane proteins exhibited a high heterogeneity on protein abundance distribution: there were many proteins identified only in one (or few) isolates, with the highest number for 857/06, followed by 41/14 (Figure 3). A qualitative analysis for enrichment of KO terms (available in Table S1) showed that the isolate 857/06 seemed to express higher levels, as well as some exclusive proteins participating in solute binding and acting as transporters (Supplementary Dataset 1). Nevertheless, most of these proteins were also found in the other strains. On the other hand, the isolate 41/14 had a higher proportion of exclusive proteins annotated as “putative membrane” or “putative exported”, without any assigned KO.

### 3.3. Prediction of New Potential Vaccine Candidates

We combined our experimental proteomics approach with bioinformatic tools to predict new vaccine candidates with antigenic potential, either as whole proteins or sequence fragments from some particular protein(s). A priori, the highly abundant and exposed proteins—mainly cell wall-anchored proteins and lipoproteins—are expected to be antigenic, as extensively demonstrated. Therefore, we searched for new PVCs in the group of membrane proteins, and particularly within those with more than one predicted TMD, as they have been poorly studied at immunogenic and protective levels.

**Figure 4:**
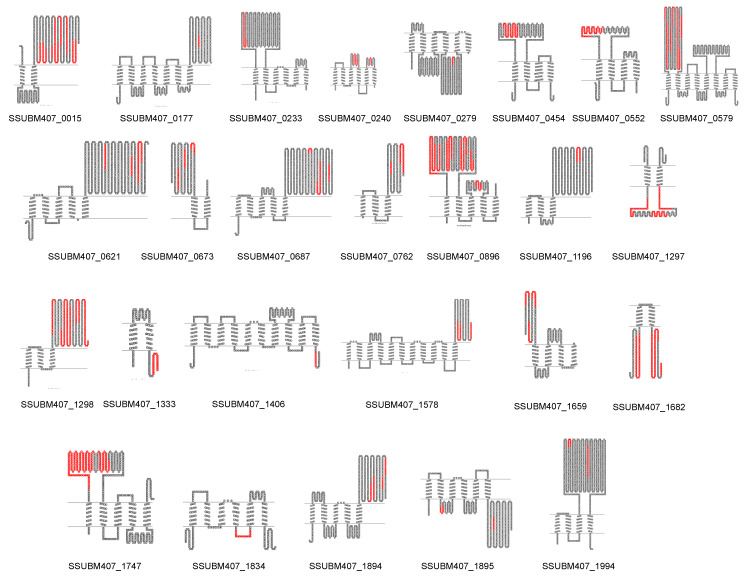
Topological representation of the 26 identified multi-transmembrane proteins in the six *Streptococcus suis* human clinical isolates. The TMHMM algorithm was used to predict transmembrane domains (TMD) after prediction of subcellular localization by LocateP v2. Sequences in red represent the peptides experimentally identified after bacterial “shaving” followed by LC-MS/MS analysis.

Then, we mapped on the protein sequences the peptides experimentally identified (Appendix A, Supplementary Dataset 2) for the 26 multi-transmembrane proteins found after “shaving” the bacteria and analyzing the “surfomes” via LC-MS/MS and represented with Topo2, the theoretical topologies, according to TMHMM predictions (Figure 4). For 19 out of the 26 proteins, the identified peptides matched in regions or loops that were predicted by TMHMM to be extracellularly exposed. The other 7 proteins—SSUBM407_0279, SSUBM407_1297, SSUBM407_1333, SSUBM407_1406, SSUBM407_1682, SSUBM407_1834, and SSUBM407_1895—were identified from peptides that theoretically mapped intracellular loops.

Finally, we evaluated the potential antigenicity of these 26 multi-transmembrane proteins using the web-based VaxiJen tool and compared for each protein the whole sequence with the sequences found after trypsinization, and/or comprising the regions between discontinuous peptides experimentally identified if they were close to each other. In most cases, the antigenic scores improved when the experimentally identified regions were selected, compared to the whole sequences of their corresponding proteins (Table 4). Particularly, for six proteins—SSUBM407_0454, SSUBM407_0552, SSUBM407_1333, SSUBM407_1682, SSUBM407_1834, and SSUBM407_1894—the algorithm VaxiJen predicted that the whole proteins were not antigenic (score < 0.4), but for four of them—SSUBM407_0454, SSUBM407_0552, SSUBM407_1834, and SSUBM407_1894—the selected peptides or regions corresponding to the sequences experimentally found by proteomics increased the score and caused them to be predicted as antigenic (score ≥ 0.4). For five out of the seven proteins in which the peptides found matched predicted intracellular loops—SSUBM407_1297, SSUBM407_1406, SSUBM407_1682, SSUBM407_1834, and SSUBM407_1895—there was an increase in the VaxiJen score after selecting the experimentally identified regions, compared to the whole protein sequences.

## 4. Discussion

In the interplay between cells and their environment, surface proteins are key molecules playing many important biological roles [40,41]. In the particular case of bacterial pathogens, many surface proteins are involved in virulence and pathogenicity; yet since they are normally exposed and in contact with elements of the host immune system, they have the highest chances of becoming effective candidates for drug and vaccine development [14,42].

In this work, we applied for the first time the “shaving” approach, successfully used by our research group in different Gram-positive bacteria [19,20,21,25,26,39], to perform a comparative proteomic analysis of six SS2 human clinical isolates. In a previous paper, we carried out a similar study in a large collection of *S. suis* clinical isolates from pigs belonging to different serotypes [25], which led to the discovery of an immunoprotective cell wall protein, namely SsnA [43,44]. These studies were followed by a comparative immunosecretomic analysis of the same isolates [27]. However, there is a lack of studies on *S. suis* human isolates aimed at comparing the proteomic profile with those of animal isolates and discovering potential vaccine candidates that can be effective in the next outbreak affecting humans. This is especially important given the increasing concern of emerging zoonoses.

Our proteomic analysis resulted in the identification of 131 surface proteins in the six human isolates. This number is very similar to our previous work on 39 swine isolates, in which 113 surface proteins were found [25]. Actually, the numbers of proteins identified per category of subcellular location were almost identical, except for membrane proteins—71 proteins with one or more TMD in this present work, compared to 54 in our previous work [25]. Cytoplasmic proteins, which can be released because of residual cell lysis, non-canonical secretion pathways, or via extracellular vesicle blebbing [45], were not considered in the downstream workflow of PVC selection. Here, we used as database the BM407 strain, which is a SS2 reference strain from humans; whereas in our previous work with animal isolates we used the swine isolate P1/7, as one of the reference strains for SS2 in pigs. Therefore, to compare both the current and the previous protein lists, we searched for the homologous proteins of BM407 in P1/7, and almost all of them share nearly 100% identity. The equivalence for both strains can be found in Table S1. Thus, out of the 131 surface proteins that we identified in the six human isolates, 15 cell wall proteins were also present in 39 pig isolates [25], as well as 28 lipoproteins, 8 secreted proteins, and 22 membrane proteins with 1 TMD, considering that only 28 were found in pigs. This is expected as these categories are highly exposed and abundant, generating many peptides. Due to this, these proteins could be considered as potential vaccine candidates for future formulations for universal vaccines to protect both humans and animals. Actually, the vast majority of these proteins were found in the six human isolates analyzed, with some exceptions; for example, the already cited cell wall putative glucan-binding surface-anchored proteins, found only in 1086/11. However, there was a higher difference in the number of common multi-transmembrane proteins between both lists: only 10 proteins out of 26 found both in human and swine isolates were common. This is also reasonable, as these proteins are more embedded into the cell wall, therefore they are less accessible and less abundant.

Noticeably, we identified the major pilus subunit, SSUBM407_0414, in the six isolates. This was a surprising and unexpected result, as Gram-positive pilus proteins have been described to be trypsin resistant, the so-called Lancefield T antigens [46]. In previous works we used unspecific proteases, like proteinase K, to get a few peptides from this family of proteins [21,38,39]. However, in the present study 30 peptides were identified for the major pilus subunit in the six strains, representing 54% coverage on the protein sequence. We ignore the reasons of the sensitivity of SSUBM407_0414 to trypsin, compared to other pilin proteins already described to be resistant to this protease.

So far, the “shaving” approach has been used to identify proteins and to compare strains in a qualitative way (absent versus present proteins). However, we have also reported that the number of identified peptides is indicative of the protein abundance, although ratios between samples cannot be calculated using this parameter [19,21,25]. Here we used the chromatography peak areas in a label-free semi-quantitative way to report protein abundance levels. We have strong evidences that this parameter is more consistent than using spectral counting or other label-free methods (Rodríguez-Ortega, unpublished results). After PCA to evaluate the contribution of surface proteins to differences among the six SS2 human isolates, we performed clustering heatmaps to get some hints on either similarities or differences of protein expression in such strains. Thus, in general, we observed that isolates 857/06, 41/14, and 117/12 had higher abundances of surface proteins for most categories, but that exhibited a high variability of membrane proteins. This can be explained assuming that these proteins are less abundant (more embedded in the surface rendering less peptides, and lower copy numbers rendering lower peptide spectral matches), since they are more difficult to identify across all the isolates, in contrast to lipoproteins or cell wall proteins. Furthermore, KEGG Mapper analysis showed that the isolates 857/06 and 41/14 expressed more solute-binding and transporter proteins. We ignore whether this can be related to biological phenomena, like higher virulence or antibiotic resistance, or not as we lack this information from the isolates.

Twenty years ago reverse vaccinology revolutionized the way to select vaccine candidates; it follows a new concept according to which the most promising antigens can be easily predicted from a genome using adequate algorithms [28,29]. The first success in applying this concept was reached with *Neisseria meningitidis* [47]. Surface proteins, in contact with the environment and, therefore, the host immune system, have the highest chances to become effective vaccine antigens. It is estimated that approximately one third of genes are encoded in any genome code for surface proteins, including those secreted to the extracellular milieu [14]. However, the genome does not inform which, when, and to what extent genes are expressed and the coded proteins synthesized. This limitation caused the concept of reverse vaccinology to move forward and include wet lab-based techniques, as protein arrays or classical proteomics [48]. In previous works, we demonstrated that “shaving”-based proteomics is useful to select the most antigenic surface proteins, as there is a strong correlation between trypsinized proteins and antibody binding to the surface of live, intact cells [21]. In this present study we found most of the surface proteins showing protective activity in animal models so far, including Sao [49,50], Sat [51], SsnA [43], HP0197 [52], SsPepO [53], and Sly [54]. We did not identify either the muramidase release factor Mrp or the extracellular protein factor Epf, two important *S. suis* protective antigens [55]. Although these two highly variable genes are present in the BM407 strain, it does not express both proteins, as many other strains belonging either to serotype 2 or to other serotypes [56]. It is therefore not surprising that the human isolates analyzed in this study do not express such proteins.

Moreover, subcellular localization algorithms based on signal features sometimes provide misleading predictions. We also demonstrated that those misleading predictions can be corrected, or at least revisited, by mapping the experimentally identified peptides on the predicted protein topologies [19,21,25,26,45]. In the present study, in the search for alternative PVCs, we focused on less considered proteins, such as multi-transmembrane proteins, since lipoproteins and cell wall proteins have been extensively analyzed in their immunogenicity and vaccine potential in animal models of infection (for an extensive review on *S. suis* protein vaccine candidates, see the review by [13]). Then, we mapped the peptides identified in our proteomic workflow on the corresponding protein sequences and represented the theoretical topologies. For most proteins (19 out of 26) there was a concordance between the regions identified and the predicted extracellular domains. Then, we used a machine-learning (ML) algorithm, VaxiJen, to predict the antigenicity of such proteins and to compare the whole protein sequences with the most restricted regions for each containing the peptides experimentally identified. We chose VaxiJen because ML-based predictors do not discard proteins based on sequence signal features as decision tree predictors do (e.g., Vaxign, Jenner-predict). Multi-transmembrane proteins would be discarded if a decision-tree algorithm was used, whereas ML-based ones consider all the proteins [57]. In our study, most of the multi-transmembrane proteins increased their antigenicity score after selecting the experimentally identified regions compared to the whole sequences; particularly, five out of the seven proteins for which there was not a concordance in topology between predicting algorithms and experimental results. This indicates that the combination of proteomics and bioinformatics within the reverse vaccinology concept is useful for selecting a priori not considered PVCs, and for refining the workflows for candidates to enter the production and test pipelines. The “shaving” approach is a reinforcement of classical reverse vaccinology only based on in silico studies of subcellular location, protein topology, and antigenicity for a more accurate selection of PVCs.

## 5. Conclusions

This study shows the first comparative “shaving”-based proteomic analysis of several serotype 2 human isolates of the major zoonotic pathogen *S. suis*. A list of proteins identified was obtained, many of them present in all or most of the strains. Multivariate classification and clustering analysis allowed us to distinguish the expression pattern and abundances of surface proteins. The combination of proteomics and bioinformatic tools made it possible to select, for further testing in animal models, PVCs that would not be prioritized in classical reverse vaccinology approaches, such as many multi-transmembrane proteins or some exposed domains of these. Thus, our approach can be considered as an experimental-aided reverse vaccinology method for PVC selection. Our study is a needed step in the further necessary workflow to measure the immunogenic and protective capacities of such selected polypeptides. Further research is thus necessary to address this point.

## Figures and Tables

**Table 1:** *Streptococcus suis* human clinical isolates used in this study.

Clinical Isolate	Province of Origin	Source
117/12	Ciudad Real	Blood
1299/06	Huelva	Cerebrospinal fluid
1086/11	Lugo	Blood
34/11	Asturias	Cerebrospinal fluid
857/06	Córdoba	Cerebrospinal fluid
41/14	Vitoria	Blood

**Table 2:** Summary of surface proteins identified in the six *Streptococcus suis* human isolates after “shaving” and LC-MS/MS analysis.

Protein Category ^a^	Number of Identified Proteins	Number of Predicted Proteins in *S. suis* BM407 Genome	Identified/Predicted (%)
Lipoprotein	31	40	77.5
Cell wall	18	20	90
Secreted	11	18	61.1
Membrane (1 TMD)	45	110	40.9
Multi-transmembrane	26	369	7.1
Total	131	557	23.5

^a^ Protein categories were defined as follows from LocateP v2 predictions: lipoproteins were those predicted as lipid-anchored proteins; cell wall proteins, as those possessing an LPXTG motif; secretory proteins, as those with an SP1-type signal peptide; membrane proteins with one transmembrane domain (TMD), as those possessing either a *C*- or an *N*-terminally anchored transmembrane region; multi-transmembrane proteins were membrane proteins with more than one TMD. The sum of the previous categories is considered as the total number of surface proteins (either identified experimentally or predicted from the *S. suis* BM407 genome).

**Table 3:** Predicted surface proteins identified in the six *Streptococcus suis* human clinical isolates after “shaving” bacterial cells and LC-MS/MS analysis.

Locus	Protein Annotation	117/12	1299/06	1086/11	34/11	857/06	41/14
Lipoproteins ^a^							
SSUBM407_0110	Zinc-binding protein AdcA	×	×	×	×	×	×
SSUBM407_0147	Putative endopeptidase	×	×		×		×
SSUBM407_0275	Extracellular solute-binding protein	×	×	×	×	×	×
SSUBM407_0359	Putative penicillin-binding protein 1A	×	×	×	×	×	×
SSUBM407_0582	Putative lipoprotein	×	×	×	×	×	×
SSUBM407_0612	Peptidyl-prolyl cis-trans isomerase	×	×	×	×	×	×
SSUBM407_0619	Extracellular solute-binding protein	×	×		×	×	×
SSUBM407_0696	Putative lipoprotein	×	×	×	×	×	×
SSUBM407_0697	Putative lipoprotein	×	×	×	×	×	×
SSUBM407_0711	Foldase protein PrsA	×	×	×	×	×	×
SSUBM407_0831	Putative phosphate ABC transporter, extracellular phosphate-binding lipoprotein	×	×			×	×
SSUBM407_0849	Putative lipoprotein	×	×	×	×	×	×
SSUBM407_0904	Putative extracellular amino acid-binding protein	×	×		×	×	×
SSUBM407_1037	Putative lipoprotein	×	×	×	×	×	×
SSUBM407_1134	Putative D-alanyl-D-alanine carboxypeptidase	×	×	×	×	×	×
SSUBM407_1174	Putative lipoprotein	×	×	×	×	×	×
SSUBM407_1218	Putative ferrichrome-binding protein	×	×	×	×	×	×
SSUBM407_1232	Putative exported protein		×				×
SSUBM407_1311	Putative amino acid ABC transporter, extracellular amino acid-binding protein	×	×	×	×	×	×
SSUBM407_1441	Branched-chain amino acid ABC transporter, amino acid-binding protein	×	×	×	×	×	×
SSUBM407_1449	Multiple sugar-binding protein	×	×	×	×	×	×
SSUBM407_1467	Streptococcal histidine triad-family protein	×	×	×	×	×	×
SSUBM407_1633	FAD:protein FMN transferase	×	×		×	×	×
SSUBM407_1634	Putative lipoprotein	×	×	×	×	×	×
SSUBM407_1651	Lipoprotein	×	×	×	×	×	×
SSUBM407_1713	Putative lipoprotein	×	×	×	×	×	×
SSUBM407_1736	Putative oligopeptide-binding protein OppA	×	×	×	×	×	×
SSUBM407_1923	Putative amino-acid ABC transporter extracellular-binding protein	×	×	×	×	×	×
SSUBM407_1939	Extracellular metal cation-binding protein	×	×	×	×	×	×
SSUBM407_1980	Maltodextrin-binding protein	×	×	×	×	×	×
SSUBM407_1998	Putative fumarate reductase flavoprotein subunit	×	×	×	×	×	×
Cell wall proteins							
SSUBM407_0180	Putative surface-anchored protein	×	×	×	×	×	×
SSUBM407_0194	Putative surface-anchored protein	×	×	×	×	×	×
SSUBM407_0244	Putative surface-anchored protein	×	×	×	×	×	×
SSUBM407_0414	Major pilus subunit	×	×	×	×	×	×
SSUBM407_0471	Putative glucan-binding surface-anchored protein			×			
SSUBM407_0574	Putative surface-anchored dipeptidase	×	×	×	×	×	×
SSUBM407_0588	Putative surface-anchored protein	×	×	×	×	×	×
SSUBM407_0646	Putative surface-anchored zinc carboxypeptidase	×	×	×	×	×	×
SSUBM407_0661	Putative surface-anchored protein	×	×	×	×	×	×
SSUBM407_0918	Putative 5′-nucleotidase	×	×	×	×	×	×
SSUBM407_0949	Putative glucan-binding surface-anchored protein			×			
SSUBM407_1432	Putative surface-anchored 5′-nucleotidase	×	×	×	×	×	×
SSUBM407_1550	Putative surface-anchored protein	×	×		×		×
SSUBM407_1830	Surface-anchored DNA nuclease	×	×	×	×	×	×
SSUBM407_1843	Putative surface-anchored serine protease	×	×	×	×	×	×
SSUBM407_1919	Putative surface-anchored amylopullulanase	×	×	×	×	×	×
SSUBM407_1949	Putative surface-anchored 2′,3′-cyclic-nucleotide 2′-phosphodiesterase	×	×	×	×	×	×
SSUBM407_0900	Putative IgA-specific zinc metalloproteinase	×	×	×	×	×	×
Multi-transmembrane proteins							
SSUBM407_0015	ATP-dependent zinc metalloprotease FtsH					×	×
SSUBM407_0177	Putative glycerophosphodiester phosphodiesterase			×			
SSUBM407_0233	Putative membrane protein						×
SSUBM407_0240	Glycerol facilitator-aquaporin					×	
SSUBM407_0279	Putative cation-transporting ATPase	×	×	×	×		
SSUBM407_0454	Putative permease	×			×		
SSUBM407_0552	Cell division protein FtsX					×	
SSUBM407_0579	ABC transporter permease protein	×	×	×	×		×
SSUBM407_0621	DNA translocase FtsK		×				
SSUBM407_0673	Putative glycosyl transferase					×	×
SSUBM407_0687	Putative sulfatase		×				×
SSUBM407_0762	Putative membrane protein	×	×		×	×	×
SSUBM407_0896	Putative glutamine ABC transporter, glutamine-binding protein/permease protein	×	×	×	×	×	×
SSUBM407_1196	Putative membrane protein						×
SSUBM407_1297	Putative chain length determinant protein	×	×		×	×	×
SSUBM407_1298	Integral membrane regulatory protein Wzg	×	×	×	×	×	×
SSUBM407_1333	Large-conductance mechanosensitive channel					×	
SSUBM407_1406	Putative peptidoglycan biosynthesis protein					×	
SSUBM407_1578	Acyltransferase family protein	×	×		×		
SSUBM407_1659	Putative mannose-specific phosphotransferase system (PTS), IID component					×	
SSUBM407_1682	Enoyl-CoA hydratase/isomerase family protein	×	×	×	×	×	×
SSUBM407_1747	Glutamine ABC transporter, glutamine-binding protein/permease protein	×	×	×	×	×	×
SSUBM407_1834	Nicotinamide mononucleotide transporter					×	
SSUBM407_1894	ABC transporter ATP-binding membrane protein					×	
SSUBM407_1895	ABC transporter ATP-binding membrane protein					×	
SSUBM407_1994	Putative beta-glucosidase			×			
Membrane proteins (1 TMD)							
SSUBM407_0010	Putative septum formation initiator protein	×					
SSUBM407_0017	Cell shape-determining protein MreC	×	×	×	×	×	×
SSUBM407_0019	Putative amidase	×	×	×	×	×	×
SSUBM407_0116	Putative penicillin-binding protein 1B	×	×	×	×	×	×
SSUBM407_0169	Putative membrane protein						×
SSUBM407_0203	Signal peptidase I	×	×		×	×	×
SSUBM407_0366	Ribonuclease Y					×	
SSUBM407_0531	Glycosyl hydrolases family protein		×	×	×	×	×
SSUBM407_0599	Sensor histidine kinase					×	
SSUBM407_0603	Penicillin-binding protein 2b	×	×		×		×
SSUBM407_0686	Streptococcal histidine triad-family protein	×	×	×	×	×	×
SSUBM407_0768	ATP synthase subunit b					×	
SSUBM407_0777	Putative competence associated endonuclease	×	×	×	×	×	×
SSUBM407_0856	Sortase SrtA	×	×	×	×	×	×
SSUBM407_1034	Putative lipoprotein			×			
SSUBM407_1039	Putative membrane protein	×	×		×		×
SSUBM407_1048	Putative membrane protein	×			×	×	
SSUBM407_1080	Spermidine/putrescine extracellular binding protein	×	×	×	×	×	×
SSUBM407_1087	Putative polysaccharide deacetylase	×	×	×	×	×	×
SSUBM407_1116	Putative exported protein						×
SSUBM407_1215	Putative acyltransferase					×	
SSUBM407_1225	Putative D-alanyl-lipoteichoic acid biosynthesis protein	×	×		×		×
SSUBM407_1315	Signal peptidase I		×		×	×	×
SSUBM407_1355	Flotillin family protein					×	
SSUBM407_1368	Putative exported protein				×		
SSUBM407_1403	Septation ring formation regulator EzrA					×	×
SSUBM407_1468	Putative membrane protein					×	×
SSUBM407_1494	Putative exported protein					×	
SSUBM407_1524	Peptidoglycan GlcNAc deacetylase	×	×	×		×	×
SSUBM407_1536	GDSL-like lipase/acylhydrolase protein	×	×	×		×	×
SSUBM407_1544	Putative neutral zinc metallopeptidase	×	×	×		×	×
SSUBM407_1564	Endopeptidase La	×	×	×		×	×
SSUBM407_1622	Putative penicillin binding protein 2x	×	×	×	×	×	×
SSUBM407_1750	Putative membrane protein					×	
SSUBM407_1847	Penicillin-binding protein 2a	×	×	×	×	×	×
SSUBM407_1955a	Putative accessory pilus subunit	×	×	×	×		×
SSUBM407_0137	Putative exported protein		×	×	×	×	×
SSUBM407_0299	Streptococcal histidine triad-family protein	×	×	×	×		×
SSUBM407_0558	Thiol-activated cytolysin (suilysin)	×	×	×	×	×	×
SSUBM407_0564	GTPase Era	×	×	×	×	×	×
SSUBM407_0596	Putative glutamine-binding protein	×	×	×	×	×	×
SSUBM407_0725	Putative gluconate 5-dehydrogenase					×	
SSUBM407_1737	Putative D-alanyl-D-alanine carboxypeptidase	×	×	×	×	×	×
SSUBM407_2015	Putative exported protein	×	×	×	×	×	×
SSUBM407_1318	Putative Mac family protein	×	×	×	×	×	×
Secreted proteins							
SSUBM407_0206	Putative exported protein	×	×	×	×	×	×
SSUBM407_0452	Putative exported protein	×	×	×	×	×	×
SSUBM407_0662	Putative N-acetylmuramoyl-L-alanine amidase	×	×	×	×	×	×
SSUBM407_1229	Putative exported protein	×	×		×	×	×
SSUBM407_1435	Putative exported protein	×	×	×	×	×	×
SSUBM407_1493	Putative exported protein					×	×
SSUBM407_1689	Putative exported protein	×	×	×	×	×	×
SSUBM407_1726	LytR family regulatory protein	×	×	×	×	×	×
SSUBM407_1898	UTP-glucose-1-phosphate uridylyltransferase	×	×	×	×	×	×
SSUBM407_1924	Putative amidase	×	×	×	×	×	×
SSUBM407_2032	Serine protease	×	×	×	×	×	×

^a^ Protein categories were defined as follows from LocateP v2 predictions: lipoproteins were those predicted as lipid-anchored proteins; cell wall proteins, as those possessing an LPXTG motif; secretory proteins, as those with an SP1-type signal peptide; membrane proteins with one transmembrane domain (TMD), as those possessing either a *C*- or an *N*-terminally anchored transmembrane region; multi-transmembrane proteins were membrane proteins with more than one TMD.

**Table 4:** Predicted antigenicity of identified multi-transmembrane proteins.

Locus	Protein Annotation	VaxiJen Score (Whole Protein Sequence)	VaxiJen Score (Selected Region)
SSUBM407_0015	ATP-dependent zinc metalloprotease FtsH	0.4769	0.4083
SSUBM407_0177	Putative glycerophosphodiester phosphodiesterase	0.5947	0.736
SSUBM407_0233	Putative membrane protein	0.5118	0.3818
SSUBM407_0240	Glycerol facilitator-aquaporin	0.4743	0.0193; 0.6665 ^a^
SSUBM407_0279	Putative cation-transporting ATPase	0.4129	−0.7033
SSUBM407_0454	Putative permease	0.3927	0.5403
SSUBM407_0552	Cell division protein FtsX	0.3305	0.5012
SSUBM407_0579	ABC transporter permease protein	0.5152	0.6518
SSUBM407_0621	DNA translocase FtsK	0.6354	0.5652; 0.0679; 0.4148 ^b^
SSUBM407_0673	Putative glycosyl transferase	0.406	0.4508; 0.4627; 1.0152 ^b^
SSUBM407_0687	Putative sulfatase	0.4886	−1.0353; 0.1934; 1.2694 ^b^
SSUBM407_0762	Putative membrane protein	0.5909	0.5467; 0.4076 ^c^
SSUBM407_0896	Putative glutamine ABC transporter, glutamine-binding protein/permease protein	0.4815	0.5710; 0.5193 ^d^
SSUBM407_1196	Putative membrane protein	0.614	1.542
SSUBM407_1297	Putative chain length determinant protein	0.429	0.5168
SSUBM407_1298	Integral membrane regulatory protein Wzg	0.4806	0.4464
SSUBM407_1333	Large-conductance mechanosensitive channel	0.1327	−0.3111
SSUBM407_1406	Putative peptidoglycan biosynthesis protein	0.4707	1.3646
SSUBM407_1578	Acyltransferase family protein	0.5404	0.6473; 0.3350 ^c^
SSUBM407_1659	Putative mannose-specific phosphotransferase system (PTS), IID component	0.5271	0.5209
SSUBM407_1682	Enoyl-CoA hydratase/isomerase family protein	0.2805	0.3215; 0.3055 ^e^
SSUBM407_1747	Glutamine ABC transporter, glutamine-binding protein/permease protein	0.4425	0.5469
SSUBM407_1834	Nicotinamide mononucleotide transporter	0.3328	1.5844
SSUBM407_1894	ABC transporter ATP-binding membrane protein	0.3206	1.0090; 0.4935; −0.0553; 0.2739 ^f^
SSUBM407_1895	ABC transporter ATP-binding membrane protein	0.4377	1.4975; 0.4104 ^g^
SSUBM407_1994	Putative beta-glucosidase	0.4664	0.5024; 0.3803 ^c^

^a^ The first score corresponds to the peptide matching the loop between the 3rd and the 4th transmembrane domain (TMD); the second score, to the peptide matching the loop between the 5th and the 6th TMD. ^b^ Scores corresponding to each of the three peptides identified in this protein, from *N*- to *C*-term. ^c^ Scores corresponding to each of the two peptides identified in this protein, from *N*- to *C*-term. ^d^ The first score corresponds to the region covering from the first to the last peptide identified matching the extracellular loop between the first and the second TMD; the second score, to the peptide matching the loop between the 3rd and the 4th TMD. ^e^ The two scores correspond to the regions covered by peptides matching the first and the second predicted intracellular loops, from *N*- to *C*-term, respectively. ^f^ Scores corresponding to each of the four peptides identified in this protein, from *N*- to *C*-term. ^g^ The two scores correspond to the peptides matching the 2nd and the 4th predicted intracellular loops, from *N*- to *C*-term, respectively.

## Supplementary Materials

The following are available online at https://www.mdpi.com/2076-393X/8/2/188/s1, Figure S1: Sequence coverage of protein SSUBM407_0414 by peptides identified after bacterial “shaving” followed by LC-MS/MS analysis; Figure S2: Euclidean distances among biological replicates of global surface proteins identified in the six *Streptococcus suis* human clinical isolates; Supplementary Table S1: List and quantitative values of identified surface proteins, as predicted by LocateP v2; Supplementary Dataset 1: Qualitative enrichment of KEGG Orthology (KO) terms; Supplementary Dataset 2: Complete list of proteins and peptides identified in the six *Streptococcus suis* human clinical isolates.

## Appendix A

The complete lists of proteins, peptides, and all raw data information for the three replicates of each of the six clinical isolates, including peak areas for further quantification, are available as supplementary material in this work as Supplementary Dataset 2.

## References

[1] Vötsch D., Willenborg M., Weldearegay Y.B., Valentin-Weigand P. (2018). Streptococcus suis—The “Two Faces” of a Pathobiont in the Porcine Respiratory Tract. Front. Microbiol..

[2] Gottschalk M., Xu J., Calzas C., Segura M. (2010). Streptococcus suis: A new emerging or an old neglected zoonotic pathogen?. Future Microbiol..

[3] Lun Z.R., Wang Q.P., Chen X.G., Li A.X., Zhu X.Q. (2007). Streptococcus suis: An emerging zoonotic pathogen. Lancet Infect. Dis..

[4] Weinert L.A., Chaudhuri R.R., Wang J., Peters S.E., Corander J., Jombart T., Baig A., Howell K.J., Vehkala M., Valimaki N. (2015). Genomic signatures of human and animal disease in the zoonotic pathogen Streptococcus suis. Nat. Commun..

[5] Kerdsin A., Takeuchi D., Nuangmek A., Akeda Y., Gottschalk M., Oishi K. (2020). Genotypic Comparison between Streptococcus suis Isolated from Pigs and Humans in Thailand. Pathogens.

[6] Nghia H.D., Ho D.T., Tu L.E., Le T.P., Wolbers M., Thai C.Q., Cao Q.T., Hoang N.V., Nguyen V.M., Nga T.V. (2011). Risk factors of Streptococcus suis infection in Vietnam. A case-control study. PLoS ONE.

[7] Takeuchi D., Kerdsin A., Pienpringam A., Loetthong P., Samerchea S., Luangsuk P., Khamisara K., Wongwan N., Areeratana P., Chiranairadul P. (2012). Population-based study of Streptococcus suis infection in humans in Phayao Province in northern Thailand. PLoS ONE.

[8] Segura M., Fittipaldi N., Calzas C., Gottschalk M. (2017). Critical Streptococcus suis Virulence Factors: Are They All Really Critical?. Trends Microbiol..

[9] Wertheim H.F., Nghia H.D., Taylor W., Schultsz C. (2009). Streptococcus suis: An emerging human pathogen. Clin. Infect. Dis..

[10] Goyette-Desjardins G., Auger J.P., Xu J., Segura M., Gottschalk M. (2014). Streptococcus suis, an important pig pathogen and emerging zoonotic agent-an update on the worldwide distribution based on serotyping and sequence typing. Emerg. Microbes Infect..

[11] Baums C.G., Kock C., Beineke A., Bennecke K., Goethe R., Schroder C., Waldmann K.H., Valentin-Weigand P. (2009). Streptococcus suis bacterin and subunit vaccine immunogenicities and protective efficacies against serotypes 2 and 9. Clin. Vaccine Immunol..

[12] Büttner N., Beineke A., de Buhr N., Lilienthal S., Merkel J., Waldmann K.H., Valentin-Weigand P., Baums C.G. (2012). Streptococcus suis serotype 9 bacterin immunogenicity and protective efficacy. Vet. Immunol. Immunopathol..

[13] Segura M. (2015). Streptococcus suis vaccines: Candidate antigens and progress. Expert Rev. Vaccines.

[14] Grandi G. (2006). Genomics and proteomics in reverse vaccines. Methods Biochem. Anal..

[15] Zagursky R.J., Anderson A.S. (2008). Application of genomics in bacterial vaccine discovery: A decade in review. Curr. Opin. Pharmacol..

[16] Adamczyk-Poplawska M., Markowicz S., Jagusztyn-Krynicka E.K. (2011). Proteomics for development of vaccine. J. Proteom..

[17] Jagusztyn-Krynicka E.K., Dadlez M., Grabowska A., Roszczenko P. (2009). Proteomic technology in the design of new effective antibacterial vaccines. Expert Rev. Proteom..

[18] Doro F., Liberatori S., Rodríguez-Ortega M.J., Rinaudo C.D., Rosini R., Mora M., Scarselli M., Altindis E., D’Aurizio R., Stella M. (2009). Surfome analysis as a fast track to vaccine discovery: Identification of a novel protective antigen for group B Streptococcus hyper-virulent strain COH_1_. Mol. Cell. Proteom..

[19] Olaya-Abril A., Gómez-Gascón L., Jiménez-Munguía I., Obando I., Rodríguez-Ortega M.J. (2012). Another turn of the screw in shaving Gram-positive bacteria: Optimization of proteomics surface protein identification in Streptococcus pneumoniae. J. Proteom..

[20] Olaya-Abril A., Jiménez-Munguía I., Gómez-Gascón L., Obando I., Rodríguez-Ortega M.J. (2015). A Pneumococcal Protein Array as a Platform to Discover Serodiagnostic Antigens Against Infection. Mol. Cell. Proteom..

[21] Rodríguez-Ortega M.J., Norais N., Bensi G., Liberatori S., Capo S., Mora M., Scarselli M., Doro F., Ferrari G., Garaguso I. (2006). Characterization and identification of vaccine candidate proteins through analysis of the group A Streptococcus surface proteome. Nat. Biotechnol..

[22] Solis N., Cain J.A., Cordwell S.J. (2016). Comparative analysis of Staphylococcus epidermidis strains utilizing quantitative and cell surface shaving proteomics. J. Proteom..

[23] Solis N., Larsen M.R., Cordwell S.J. (2010). Improved accuracy of cell surface shaving proteomics in Staphylococcus aureus using a false-positive control. Proteomics.

[24] Solis N., Parker B.L., Kwong S.M., Robinson G., Firth N., Cordwell S.J. (2014). Staphylococcus aureus surface proteins involved in adaptation to oxacillin identified using a novel cell shaving approach. J. Proteome Res..

[25] Gómez-Gascón L., Luque I., Olaya-Abril A., Jiménez-Munguía I., Orbegozo-Medina R.A., Peralbo E., Tarradas C., Rodríguez-Ortega M.J. (2012). Exploring the pan-surfome of Streptococcus suis: Looking for common protein antigens. J. Proteom..

[26] Olaya-Abril A., Jiménez-Munguía I., Gómez-Gascón L., Obando I., Rodríguez-Ortega M.J. (2013). Identification of potential new protein vaccine candidates through pan-surfomic analysis of pneumococcal clinical isolates from adults. PLoS ONE.

[27] Gómez-Gascón L., Luque I., Tarradas C., Olaya-Abril A., Astorga R.J., Huerta B., Rodríguez-Ortega M.J. (2018). Comparative immunosecretome analysis of prevalent Streptococcus suis serotypes. Comp. Immunol. Microbiol. Infect. Dis..

[28] Rappuoli R. (2000). Reverse vaccinology. Curr. Opin. Microbiol..

[29] Rappuoli R. (2001). Reverse vaccinology, a genome-based approach to vaccine development. Vaccine.

[30] Rodríguez-Ortega M.J. (2018). “Shaving” Live Bacterial Cells with Proteases for Proteomic Analysis of Surface Proteins. Methods Mol. Biol..

[31] UniProt. https://www.uniprot.org/proteomes/UP000009077.

[32] Zhou M., Boekhorst J., Francke C., Siezen R.J. (2008). LocateP: Genome-scale subcellular-location predictor for bacterial proteins. BMC Bioinform..

[33] Krogh A., Larsson B., von Heijne G., Sonnhammer E.L. (2001). Predicting transmembrane protein topology with a hidden Markov model: Application to complete genomes. J. Mol. Biol..

[34] Almagro Armenteros J.J., Tsirigos K.D., Sonderby C.K., Petersen T.N., Winther O., Brunak S., von Heijne G., Nielsen H. (2019). SignalP 5.0 improves signal peptide predictions using deep neural networks. Nat. Biotechnol..

[35] Juncker A.S., Willenbrock H., Von Heijne G., Brunak S., Nielsen H., Krogh A. (2003). Prediction of lipoprotein signal peptides in Gram-negative bacteria. Protein Sci..

[36] Kanehisa M., Sato Y. (2020). KEGG Mapper for inferring cellular functions from protein sequences. Protein Sci..

[37] Doytchinova I.A., Flower D.R. (2007). VaxiJen: A server for prediction of protective antigens, tumour antigens and subunit vaccines. BMC Bioinform..

[38] Garibaldi M., Rodríguez-Ortega M.J., Mandanici F., Cardaci A., Midiri A., Papasergi S., Gambadoro O., Cavallari V., Teti G., Beninati C. (2010). Immunoprotective activities of a Streptococcus suis pilus subunit in murine models of infection. Vaccine.

[39] Rodríguez-Ortega M.J., Luque I., Tarradas C., Bárcena J.A. (2008). Overcoming function annotation errors in the Gram-positive pathogen Streptococcus suis by a proteomics-driven approach. BMC Genom..

[40] Navarre W.W., Schneewind O. (1999). Surface proteins of gram-positive bacteria and mechanisms of their targeting to the cell wall envelope. Microbiol. Mol. Biol. Rev..

[41] Solis N., Cordwell S.J. (2011). Current methodologies for proteomics of bacterial surface-exposed and cell envelope proteins. Proteomics.

[42] Rinaudo C.D., Telford J.L., Rappuoli R., Seib K.L. (2009). Vaccinology in the genome era. J. Clin. Investig..

[43] Gómez-Gascón L., Cardoso-Toset F., Amarilla P.S., Tarradas C., Carrasco L., Olaya-Abril A., Jiménez-Munguía I., Rodríguez-Ortega M.J., Luque I. (2014). A new recombinant SsnA protein combined with aluminum hydroxide protects mouse against Streptococcus suis. Vaccine.

[44] Gómez-Gascón L., Cardoso-Toset F., Tarradas C., Gómez-Laguna J., Maldonado A., Nielsen J., Olaya-Abril A., Rodríguez-Ortega M.J., Luque I. (2016). Characterization of the immune response and evaluation of the protective capacity of rSsnA against Streptococcus suis infection in pigs. Comp. Immunol. Microbiol. Infect. Dis..

[45] Olaya-Abril A., Jiménez-Munguía I., Gómez-Gascón L., Rodríguez-Ortega M.J. (2014). Surfomics: Shaving live organisms for a fast proteomic identification of surface proteins. J. Proteom..

[46] Mora M., Bensi G., Capo S., Falugi F., Zingaretti C., Manetti A.G., Maggi T., Taddei A.R., Grandi G., Telford J.L. (2005). Group A Streptococcus produce pilus-like structures containing protective antigens and Lancefield T antigens. Proc. Natl. Acad. Sci. USA.

[47] Pizza M., Scarlato V., Masignani V., Giuliani M.M., Arico B., Comanducci M., Jennings G.T., Baldi L., Bartolini E., Capecchi B. (2000). Identification of vaccine candidates against serogroup B meningococcus by whole-genome sequencing. Science.

[48] Mora M., Telford J.L. (2010). Genome-based approaches to vaccine development. J. Mol. Med..

[49] Li Y., Gottschalk M., Esgleas M., Lacouture S., Dubreuil J.D., Willson P., Harel J. (2007). Immunization with recombinant Sao protein confers protection against Streptococcus suis infection. Clin. Vaccine Immunol..

[50] Li Y., Martinez G., Gottschalk M., Lacouture S., Willson P., Dubreuil J.D., Jacques M., Harel J. (2006). Identification of a surface protein of Streptococcus suis and evaluation of its immunogenic and protective capacity in pigs. Infect. Immun..

[51] Mandanici F., Gómez-Gascón L., Garibaldi M., Olaya-Abril A., Luque I., Tarradas C., Mancuso G., Papasergi S., Bárcena J.A., Teti G. (2010). A surface protein of Streptococcus suis serotype 2 identified by proteomics protects mice against infection. J. Proteom..

[52] Zhang A., Chen B., Li R., Mu X., Han L., Zhou H., Chen H., Meilin J. (2009). Identification of a surface protective antigen, HP0197 of Streptococcus suis serotype 2. Vaccine.

[53] Li J., Xia J., Tan C., Zhou Y., Wang Y., Zheng C., Chen H., Bei W. (2011). Evaluation of the immunogenicity and the protective efficacy of a novel identified immunogenic protein, SsPepO, of Streptococcus suis serotype 2. Vaccine.

[54] Jacobs A.A., van den Berg A.J., Loeffen P.L. (1996). Protection of experimentally infected pigs by suilysin, the thiol-activated haemolysin of Streptococcus suis. Vet. Rec..

[55] Wisselink H.J., Vecht U., Stockhofe-Zurwieden N., Smith H.E. (2001). Protection of pigs against challenge with virulent Streptococcus suis serotype 2 strains by a muramidase-released protein and extracellular factor vaccine. Vet. Rec..

[56] de Greeff A., Wisselink H.J., de Bree F.M., Schultsz C., Baums C.G., Thi H.N., Stockhofe-Zurwieden N., Smith H.E. (2011). Genetic diversity of Streptococcus suis isolates as determined by comparative genome hybridization. BMC Microbiol..

[57] Dalsass M., Brozzi A., Medini D., Rappuoli R. (2019). Comparison of Open-Source Reverse Vaccinology Programs for Bacterial Vaccine Antigen Discovery. Front. Immunol..

