# Supplementary material for: Proteomic and Bioinformatic Analysis of Streptococcus suis Human Isolates: Combined Prediction of Potential Vaccine Candidates
Source: Vaccines (Basel). 2020 Apr 18;8(2):188. doi: 10.3390/vaccines8020188 (PMC7348792; doi:10.3390/vaccines8020188)
Supplement: Supplementary file 1 [file vaccines-08-00188-s001.zip › vaccines-772002 supplementary for proof/Suppl material.docx]

Supplementary Materials 1


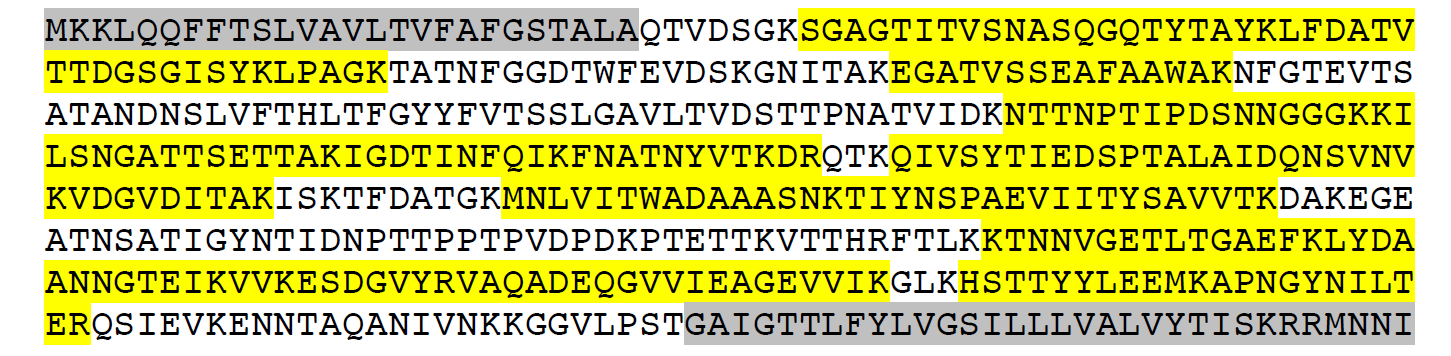


**Figure S1.** Sequence coverage of protein SSUBM407_0414 by peptides identified after bacterial “shaving” followed by LC-MS/MS analysis. Yellow highlighting represents the coverage by identified peptides. Grey highlighting indicates the sequences removed in the mature protein form.


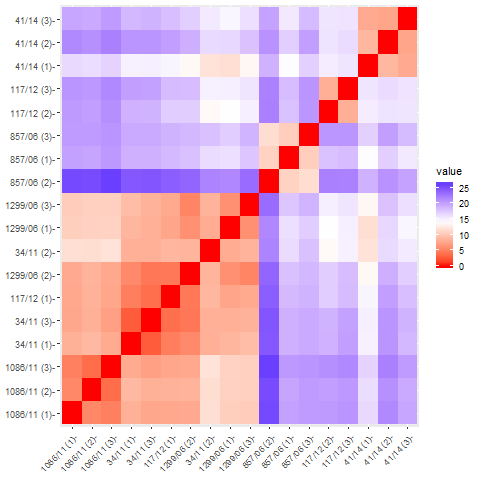


**Figure S2.** Euclidean distances among biological replicates of global surface proteins identified in the six *Streptococcus suis* human clinical isolates. The lower the value is, the lower the difference between two given samples is.
